# DNA damage and oxidative stress in human cells infected by *Trypanosoma cruzi*

**DOI:** 10.1371/journal.ppat.1009502

**Published:** 2021-04-07

**Authors:** Pilar T. V. Florentino, Davi Mendes, Francisca Nathalia L. Vitorino, Davi J. Martins, Julia P. C. Cunha, Renato A. Mortara, Carlos F. M. Menck

**Affiliations:** 1 Department of Microbiology, Institute of Biomedical Sciences, University of São Paulo, São Paulo, Brazil; 2 Special Laboratory of Cell Cycle, Butantan Institute, São Paulo, Brazil; 3 Department of Microbiology, Imunology & Parasitology, Escola Paulista de Medicina Federal University of São Paulo, São Paulo, Brazil; University of Glasgow, UNITED KINGDOM

## Abstract

*Trypanosoma cruzi* is the etiologic agent of Chagas’ disease. Infected cells with *T*. *cruzi* activate several responses that promote unbalance of reactive oxygen species (ROS) that may cause DNA damage that activate cellular responses including DNA repair processes. In this work, HeLa cells and AC16 human cardiomyocyte cell line were infected with *T*. *cruzi* to investigate host cell responses at genome level during parasites intracellular life cycle. In fact, alkaline sensitive sites and oxidized DNA bases were detected in the host cell genetic material particularly in early stages of infection. These DNA lesions were accompanied by phosphorylation of the histone H2Ax, inducing γH2Ax, a marker of genotoxic stress. Moreover, Poly [ADP-ribose] polymerase-1 (PARP1) and 8-oxoguanine glycosylase (OGG1) are recruited to host cell nuclei, indicating activation of the DNA repair process. In infected cells, chromatin-associated proteins are carbonylated, as a possible consequence of oxidative stress and the nuclear factor erythroid 2–related factor 2 (NRF2) is induced early after infection, suggesting that the host cell antioxidant defenses are activated. However, at late stages of infection, NRF2 is downregulated. Interestingly, host cells treated with glutathione precursor, N-acetyl cysteine, NRF2 activator (Sulforaphane), and also Benznidonazol (BNZ) reduce parasite burst significantly, and DNA damage. These data indicate that the balance of oxidative stress and DNA damage induction in host cells may play a role during the process of infection itself, and interference in these processes may hamper *T*. *cruzi* infection, revealing potential target pathways for the therapy support.

## Author summary

Chagas’ disease is a neglected disease that afflicts over eight million individuals worldwide. Oxidative stress is an important cell response when infected by *Trypanosoma cruzi*, etiologic agent of Chagas disease, although its role in host cell DNA/RNA metabolism has not been fully described. In this study, we investigated how *T*. *cruzi* infection and oxidative stress affect DNA molecule and the host cell metabolism. Data show that *T*. *cruzi* infection promote oxidative stress, which induce protein oxidation and DNA damage in human cells. Interestingly, control of the oxidative stress reduces the parasite capacity of infection. Until now, drug treatment with Nifurtimox or Benznidazole (BNZ) is only effective in the acute phase of the disease. This work revealed that pre administered BNZ to the host cells was also efficient in reducing oxidative stress and DNA damage, caused by parasite infection. Therefore, BNZ could be preparing cellular metabolism to respond to *T*. *cruzi* infection also by controlling the oxidative stress that may be necessary for the parasite propagation.

## Introduction

Chagas’ disease is caused by flagellated protozoan *Trypanosoma cruzi* and is endemic in 22 countries in Latin America. It is estimated that approximately 8 million individuals are infected worldwide, with 10.000 death per year [1]. The migratory flux of infected patients from Latin America to North America, Europe, Oceania, and Asia has alerted authority to a worldwide problem [2]. *T*. *cruzi* is a digenetic organism that alters its life cycle between mammal hosts and triatomine insects and presents four morphological stages: epimastigotes, metacyclic trypomastigotes, bloodstream trypomastigotes and amastigotes.

*T*. *cruzi* pathology in host mammal presents two characteristic phases. During the acute phase, which can last 4–8 weeks, infected individuals show diffuse symptoms as fever and body ache. Chronic phase presents itself after 30–40 years later and about 30–40% of the infected individuals develop severe cardiac or digestive complications [3,4]. Benznidazole (BNZ; N-benzyl-2-nitroimidazole acetamide) and Nifurtimox (NFX) are the only drugs approved to treat Chagas disease. These drugs help to clear parasites in a significant number of chronic patients, without, necessarily, restoring complications of the infection. Currently, BNZ is the only drug available in most Latin American countries and is considered to be better tolerated by patients [5,6]. BNZ is a well-established trypanocide drug that forms free radicals and electrophilic metabolites that can bind to parasite macromolecules and also oxidizes *T*. *cruzi* DNA, eventually leading to double-strand breaks in the genome of the parasite [7,8]. Although the effort to comprehend how this drug act on parasite’s physiology, there is still little understanding of the mechanism of action on host cells from patients. It is well known that patients treated with BNZ can develop serious adverse reactions, including agranulocytosis, sore throat fever, septicemia, and hepatic intolerance [6,9]. Also, BNZ can induce an imbalance of intracellular reactive oxygen species (ROS) in hepatic cells [10,11]. Recent work found that after 3 h of treatment, BNZ increases intracellular ROS, consenquently leading to an antioxidant response, mediated by nuclear factor erythroid 2-related factor 2 (NRF2), in HEPG2 cells [11].

The intracellular parasite life cycle begins when trypomastigotes invade mammalian host cells and then differentiate into the amastigote form. Intracellular amastigotes replicate into cell cytoplasm, and after cytosol is filled with parasites, they redifferentiate into trypomastigotes and disrupt host cells to infect neighboring cells [12]. *T*. *cruzi* invading mechanism generally involves an increase of Ca^2+^ influx [13] and an exacerbated production of ROS in the host cells [14–17].

It has been demonstrated that macrophage presents higher levels of NADPH oxidase (NOX) when infected with *T*. *cruzi*, resulting in ROS increase in the host cell and playing an important role in infection [18,19]. Moreover, macrophage and neutrophil that infiltrate chagasic heart produces elevated levels of ROS, NOX, and myeloperoxidase in the acute phase of Chagas’ disease [20]. During infection, mitochondrial integrity of *T*. *cruzi*-infected cardiomyocytes is affected, contributing to the inefficiency of the electron transport chain and mitochondrial membrane potential loss that also elevates intracellular O_2_^-^ [21]. Recently, Paiva *et al*. [22] proposed a novel role for ROS during infection. Instead of killing *T*. *cruzi* during infection, ROS could benefit parasite growth. The authors observed that antioxidant mechanisms mediated by NRF2 and Heme Oxygenase 1 (HO-1) were able to reduce parasite burst in macrophage, indicating that oxidative stress-mediated by infection facilitates parasite survival in host cell environment.

The interface between the elevated levels of ROS and the mechanisms involved in the parasite survival and dissemination has not yet been fully understood. *T*. *cruzi* displays many strategies to overcome ROS, with unique antioxidant enzymes and co-factors (such as, tryparedoxin peroxidase, TcSOD and trypanothione) and efficient DNA repair pathways, features that could facilitate parasite survival in mammalian cell hostile environment [23,24]. From host cell perspectives, ROS liberated in response to *T*. *cruzi* infection causes DNA damage such as 8-oxodeoxyguanine (8-oxodG) lesions and DNA strand breaks, which signal Protein poly ADP-ribosylation (PARylation) [25]. PARylation events could mediate transcription factor NFkB and, subsequentely resulting in the production of pro-inflammatory cytokines, such as TNF-α e IL-1β. More recently, the same group demonstrated that PARP-1/PAR was associated with the mitochondrial polymerase γ and mtDNA in infected cells promoting mtDNA loss. Also, inhibition of PARP1 preserves mtDNA in cardiac cells and heart of chronically infected mice, which could be beneficial to the host [26].

In this work, we propose that ROS mediated by *T*. *cruzi* infection could not only benefit parasite survival but also cause DNA damage, interfere with host cell cycle affecting DNA metabolism and RNA transcription in early and late stages of *in vitro* infection, conducting host cell death. Pretreatment of host cells with BNZ was also investigated concerning its effects on oxidative stress and DNA damage in infected cells.

## Results

### *T*. *cruzi* induces genotoxic effects in human cells and recruits base excision repair proteins at early and late stages of infection

**Fig 1:**
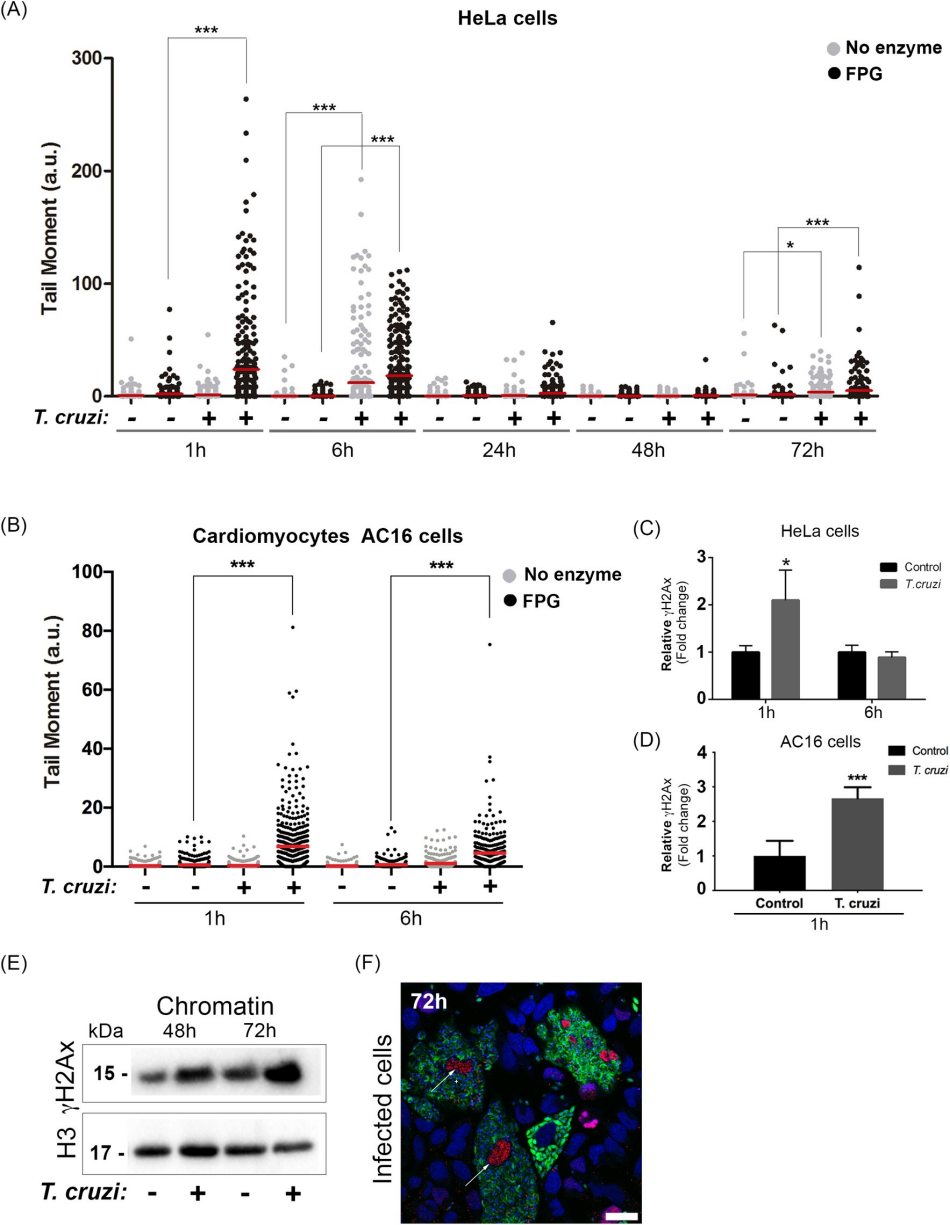
*Trypanosoma cruzi* induces DNA lesions at early and late stages of infection. Comet assay was performed in HeLa cells infected (+) or not (-) with trypomastigotes. Cells nuclei were treated with Formamidopyrimidine DNA glycosylase enzyme (FPG; black dots) or not (No enzyme; grey dots). In (A) tail moment quantification (ratio of the tail by the head length) 1 h, 6 h, 24 h, 48 h, and 72 h infected HeLa cells and (B) 1 h and 6 h AC16 cells post-infection. It was performed 3 independent experiments and at least 100 cells were analyzed per experiment; The mean is represented the red line in the graph. *p<0.05, ***p<0.001. H2Ax phosphorylated (γH2Ax) was assessed by flow cytometry after (C) 1 h and 6 h of infected HeLa cells and (D) 1 h infected AC16 cells. The graph represents a relative increase in mean fluorescence from infected cells (*T*. *cruzi*) compared to uninfected cells (control). Three independent experiments in duplicate were performed. Bars represent the mean and standard deviation. (E) Isolated chromatin from infected cells (+) or not (-) (48 h and 72 h) were immunoblotted with anti-γH2Ax. Anti-histone 3 (H3) was used as a loading control. (F) Infected cells with *T*. *cruzi-*GFP (green) after 72 h were fixed with 4% paraformaldehyde (PFA) and stained with mAb yH2Ax (red). Nuclei were stained with DAPI probe (blue). Scale bar 10 μm.

First, the induction of DNA lesions in host cells’ genome after *T*. *cruzi* infection was investigated. Breaks (alkaline sensitive sites) and oxidized base lesions, sensitive to Formamidopyrimidine DNA glycosylase (FPG) enzyme, were investigated using comet assays. Basically, higher tail moments were detected 1 h post-infection when FPG was added to the assay, indicating increased oxidized bases in the HeLa cells genome (Fig 1A). After 6 h, tail moment from infected cells was also high, almost independently on the use of FPG, indicating that, at this stage, not only FPG sensitive sites, but other types of DNA damage (single-strand breaks and alkaline sensitive sites) accumulate in the phosphodiester chain. At longer times, 24 h and 48 h, after infection, there is a clear reduction of the tail moment, indicating that DNA repair responds to the initial damage. Curiously, a few breaks are again observed in the host cells 72 h post-infection, mostly independent of FPG enzyme use. Furthermore, we assessed DNA lesions in AC16 cardiomyocyte human cell line during infection at early time points (Fig 1B). After 1 h and 6 h, we observed that *T*. *cruzi* induces a similar DNA lesion pattern found in HeLa cells (Fig 1B).

Next, the phosphorylation of H2Ax (γH2Ax), an epigenetic marker of genotoxic stress, in the early stages of infection was evaluated. A 2-fold increase in γH2Ax after 1 h of infection was observed by flow cytometry in Hela cells (Fig 1C) and AC16 cells (Fig 1D). After 6 h, phosphorylation levels were reestablished similar to uninfected cells, indicating infected HeLa cells (Fig 1C) and AC16 cells (S1 Fig) are able to deal with such stress. γH2Ax expression was also detected by western blotting in host cell chromatin, later times after infection (48 h and 72 h) (Fig 1E). γH2ax levels were higher in infected cells when compared to control cells 72 h post infection (2.18 fold change). Also, the distribution of γH2Ax assessed by immunofluorescence was found to be intense (red) at the nuclei (in blue) of HeLa cells 72 h post-infection with *T*. *cruzi* (in green) (Fig 1F).

**Fig 2:**
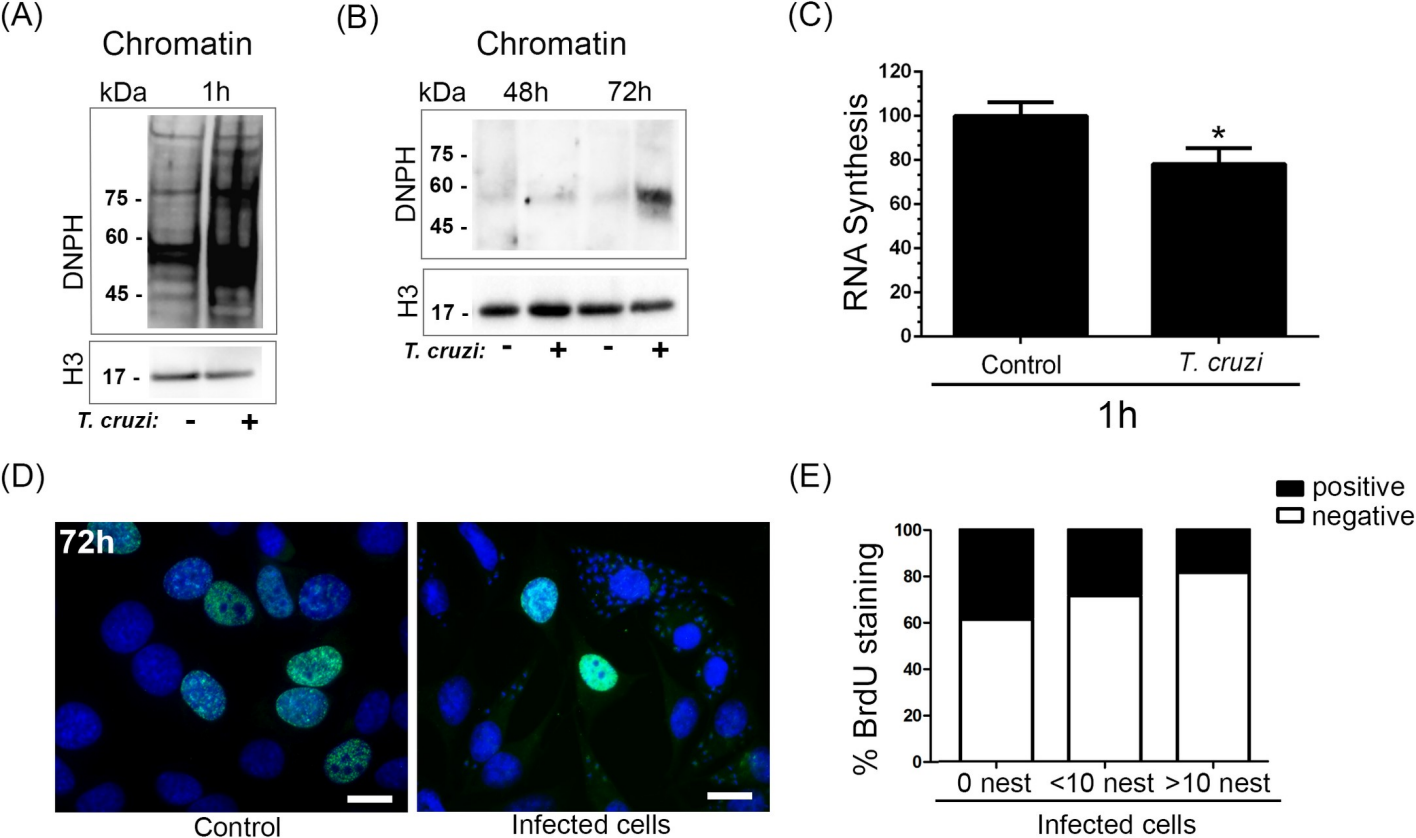
HeLa cell DNA damage generated by *Trypanosoma cruzi* affects translational and replicative machinery. (A) Isolated chromatin from infected HeLa cells (+) and control or not (-) after (A) 1 h and (B) 48 h and 72 h were immunoblotted with anti-DNPH (reveals carbonylated proteins). Anti-histone 3 (H3) was used as a loading control. (C) RNA synthesis was observed by 5-Ethynyl Uridine (EU) incorporation in infected HeLa cells or not with *T*. *cruzi* after 1 h of infection. (D) population of infected cells or not (control) were incubated with Bromodeoxyuridine (BrdU) and then stained with anti-BrdU (green). Nuclei were stained with DAPI probe (blue). (E) bars represent the percentage of stained (positive) or not (negative) cells anti-BrdU. Three independent experiments were performed. In the Infected population, it was counted cells without *T*. *cruzi* (N.I.), cells with a maximum of 10 parasites (<10), and cells with more than 10 parasites (>10).

Moreover, an increased level of carbonylated proteins present in the chromatin from infected cells was observed 1 h after infection (Fig 2A), which can be related to ROS’s presence in the host cell nucleus. Besides, higher carbonylated protein levels were detected in chromatin from infected HeLa cells compared to non-infected cells after 72 h of infection (Fig 2B). Host RNA transcription was also reduced (by close to 20%) 1 h after infection, as detected by the incorporation of 5-Ethynyl Uridine (EU) (Fig 2C). EU staining in infected and control cells by fluorescence microscopy is shown in S2 Fig. Next, the frequency of cells replicating their DNA was affected at later stages of infection. Therefore, S-phase nuclei were quantified for BrdU incorporation (Fig 2D). We observed that the higher the number of parasites in the cell, the higher the reduction of DNA synthesis, possibly due to the formation of replicative stress in cells (Fig 2E). Another possible explanation, however, could be simply due to the replication of the parasites that would deplete the available nucleotide pool in the cells. Notably, control cells presented a decrease in S-phase compared to the cells-not-containing parasite in the infected population.

**Fig 3:**
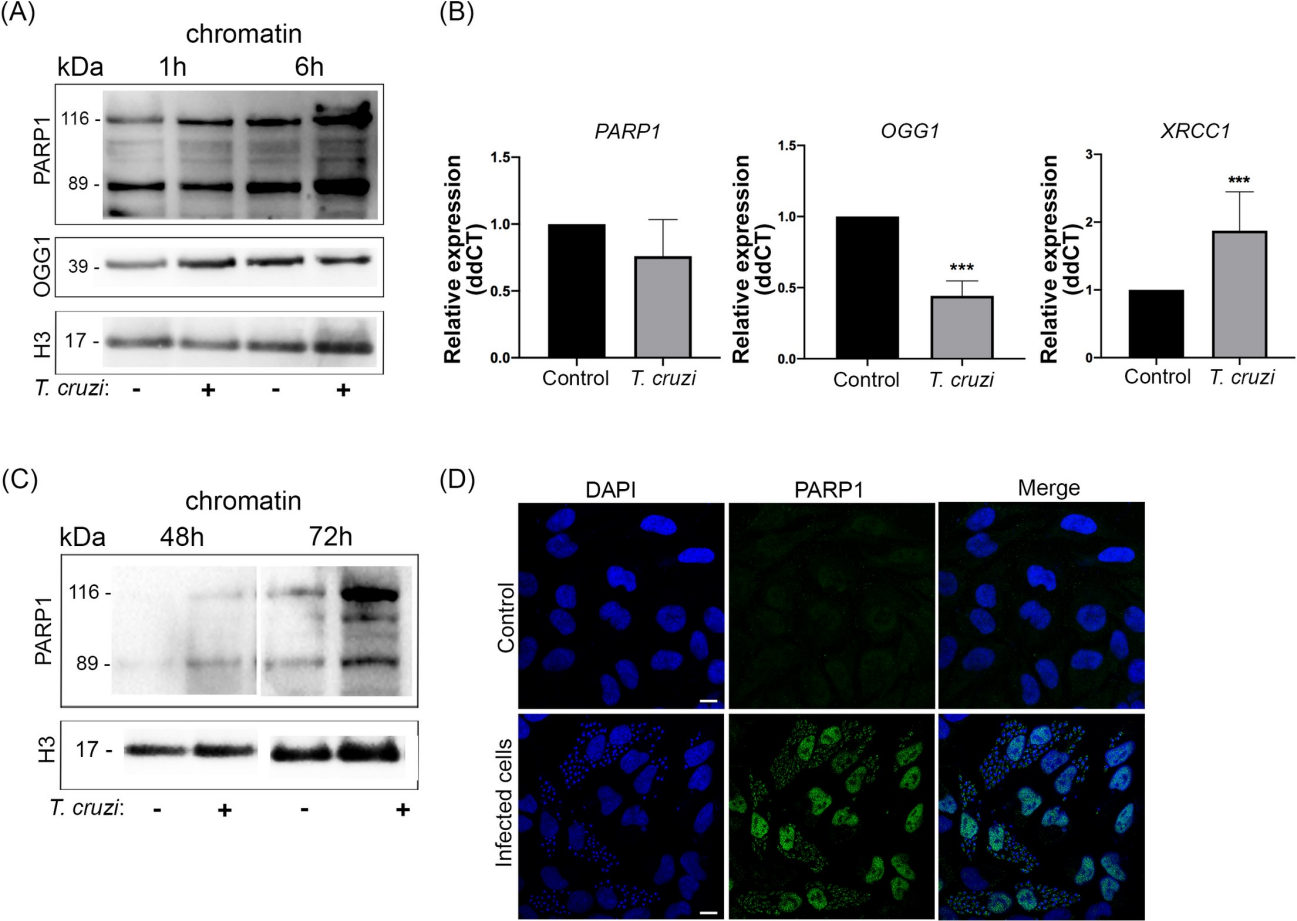
mRNA expression and recruitment of DNA repair enzymes from HeLa cells are regulated by *Trypanosoma cruzi* infection. (A) Isolated chromatin from infected cells (+) or not (-), 1 h, and 6 h post-infection were immunoblotted with anti-PARP and anti-OGG1. Anti-histone 3 (H3) was used as a loading control. (B) Relative expression (ddCT) from gene*s PARP1*, *OGG1* and *XRRC1* from infected HeLa cells compared to control 6 h post-infection. *ACTB* gene was used as the endogenous control. ***p<0.001. (C) Isolated chromatin from infected cells (+) or not, 48 h and 72 h post-infection were immunoblotted with anti-PARP1 and loading control (H3). (D) Immunofluorescence of infected cells after 72 h of infection stained with anti-PARP1 (green) and DAPI probe (blue). Scale bar: 10 μm.

As oxidized base damage and breaks were observed in infected cells, the recruitment of poly (ADP-ribose) polymerase 1 (PARP1) to host cell chromatin was investigated. PARP1 is known to be involved in the base excision repair (BER) pathway of these DNA lesions. At the early stages of infection, western blotting revealed that PARP1 is, in fact, targeted to the chromatin of infected cells 1 h (1.85 fold change) and 6 h (2.40 fold change) after infection (Fig 3A). Another BER-related protein, 8-oxoguanine glycosylase (OGG1), was associated with host cell chromatin 1 h after infection (1.80 fold change) (Fig 3A). After 6 h, however, the levels of OGG1 protein recruited in the chromatin of host cells were reestablished similar to controls (0.84 fold change). We also investigated the mRNA relative expression of PARP-1, OGG1, and XRCC1, a protein involved in repairing DNA single-strand breaks, after 6 h of infection (Fig 3B). At this time point, infected HeLa cells showed no differences in PARP-1 mRNA expression compared to control. OGG1 revealed a lower expression in infected cells. However, XRCC1 mRNA expression was higher in infected cells. In AC16 cells, PARP-1 and OGG1 kept the same expression levels compared to control (S3 Fig). Therefore, XRCC1 mRNA levels were higher in infected cells. At later stages (72 h), when cells are filled with amastigotes, an increased interaction of PARP1 (2.49 fold change) to host cell chromatin was also observed (Fig 3C). Immunostaining also confirmed a remarkable increase of PARP1 in the nuclei of infected cells (Fig 3D).

### NRF2 is differently modulated during infection

**Fig 4:**
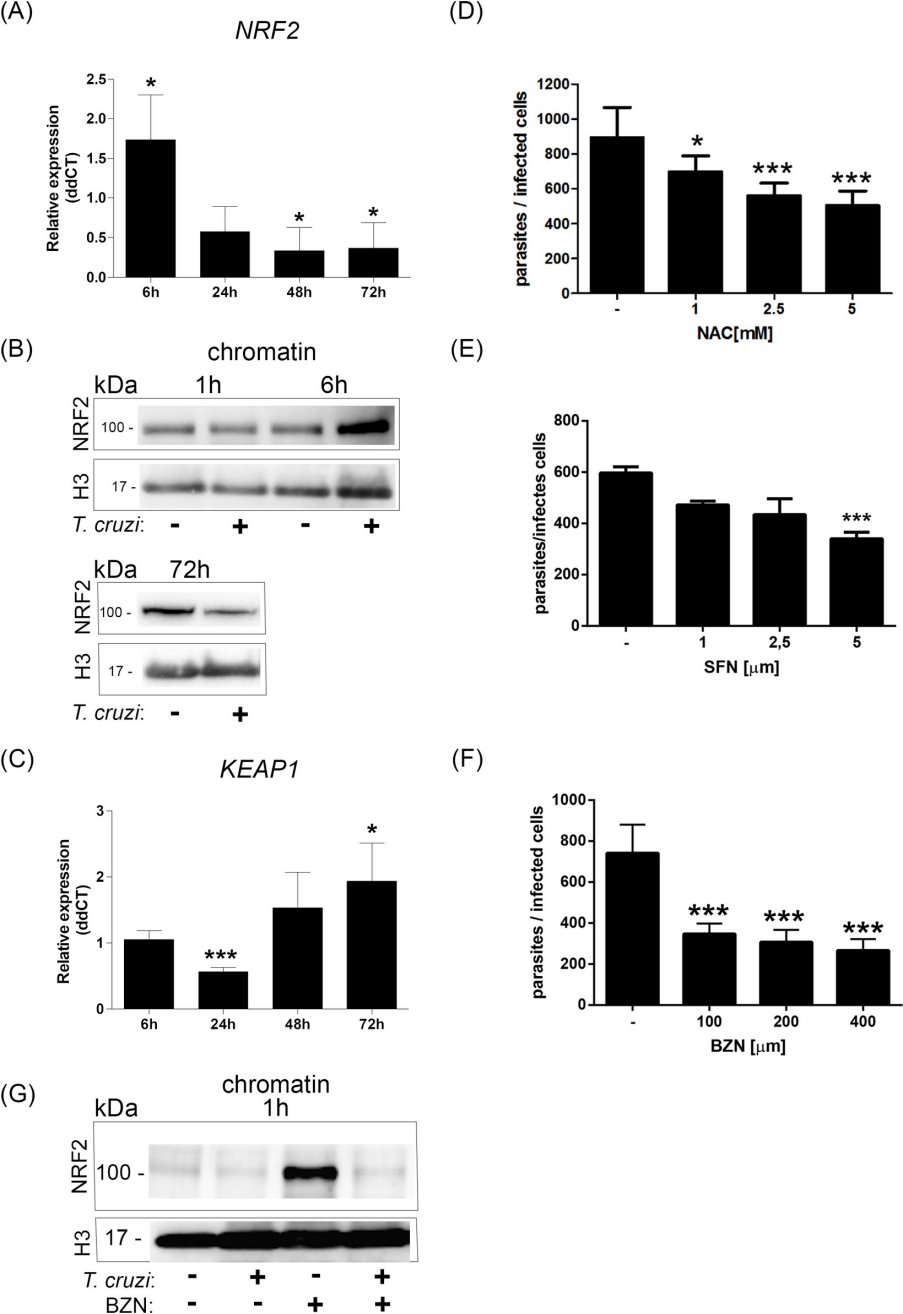
Early NRF2 activation is not sustained during infection. (A) Relative expression (ddCT) from gene *NRF2* from infected cells compared to control 6 h, 24 h, 48 h, and 72 h post-infection (p.i.). *ACTB* gene was used as the endogenous control. The bar graph represents the mean and standard deviation of mRNA relative expression of infected cells compared to the control; *p<0.05. (B) Isolated chromatin from infected (+) or control cells (-) 1 h, 6 h, or 72 h p.i. were immunoblotted with anti-NRF2. The loading control was assessed by anti-histone 3 (H3). (C) Gene *KEAP1* ddCT from infected cells compared to control 6 h, 24 h, 48 h, and 72 h p.i. *ACTB* gene was used as the endogenous control. The bar graph represents the mean and standard deviation of the mRNA relative expression of infected cells compared to the control; *p<0.05;***p<0.001. HeLa cells were infected with *T*. *cruzi* (MOI:20:1). After infection, cells were incubated with (D) N-Acetyl-L-cysteine (NAC; 1 mM, 2.5 mM, and 5 mM), (E) Sulforaphane (SFN; 1μM, 2.5 μM, and 5 μM), or (F) Benznidazole (BNZ; 100 μm, 200 μm and 400 μm). After 48 h, cells were fixed and stained. Parasites from 100 infected cells were counted per experiment. Three independent experiments were performed in duplicate or triplicate. *p<0.05; ***p<0.001. (G) Isolated chromatins from infected cells or not that were pretreated or not with BNZ (200μm) were immunoblotted with anti-NRF2. Histone 3 was used as a loading control.

As DNA base oxidation and chromatin proteins appear to be carbonylated soon after infection, the host cells are probably suffering from oxidative stress. NRF2 is a master transcription factor that controls many of the cells’ antioxidant responses. In fact, the NRF2 mRNA relative expression was higher at 6 h after infection compared to control cells (Fig 4A). Also, higher amounts of NRF2 protein were detected associated with the chromatin of infected cells compared to uninfected control cells 6 h post-infection (2.53 fold change), but not 1 h (Fig 4B).

Interestingly, NRF2 mRNA relative expression (Fig 4A) was reduced in the host cells at later times (>24 h) after infection. For protein, NRF2 protein levels associated with chromatin were even lower 72 h after infection than control cells (Fig 4B). On the other hand, the expression of KEAP1 mRNA, which interacts and negatively regulates NRF2, has an initial decrease in expression (24 h) compared to control cells and is increased later during infection (72 h) (Fig 4C). These data indicate that oxidative stress generated by parasite infection may induce NRF2 association with host cell chromatin. Still, in the final stages of infection, KEAP1 may downregulate NRF2, decreasing the association with chromatin.

In the present study, NRF2 expression was lower at later stages of infection than control cells, indicating a clear unbalanced oxidative stress in infected cells. To check the relevance of this oxidative stress in the parasite process of infection, the host cells were treated with the glutathione precursor, N-acetyl cysteine (NAC) (Fig 4D), or NRF2 inductor (sulforaphane; SFN) (Fig 4E). In both cases, NAC and SFN inhibited the growth of intracellular parasites. BNZ, an important pharmaceutical used to control trypanosome infection, also reduced intracellular amastigote multiplication in infected cells (Fig 4F). Interestingly, cells pretreated with BNZ also result in NRF2 recruited in the chromatin, although when cells are infected, the levels of NRF2 are highly reduced (Fig 4G).

### Benznidazole treatment induces an adaptative response to DNA generated injuries

**Fig 5:**
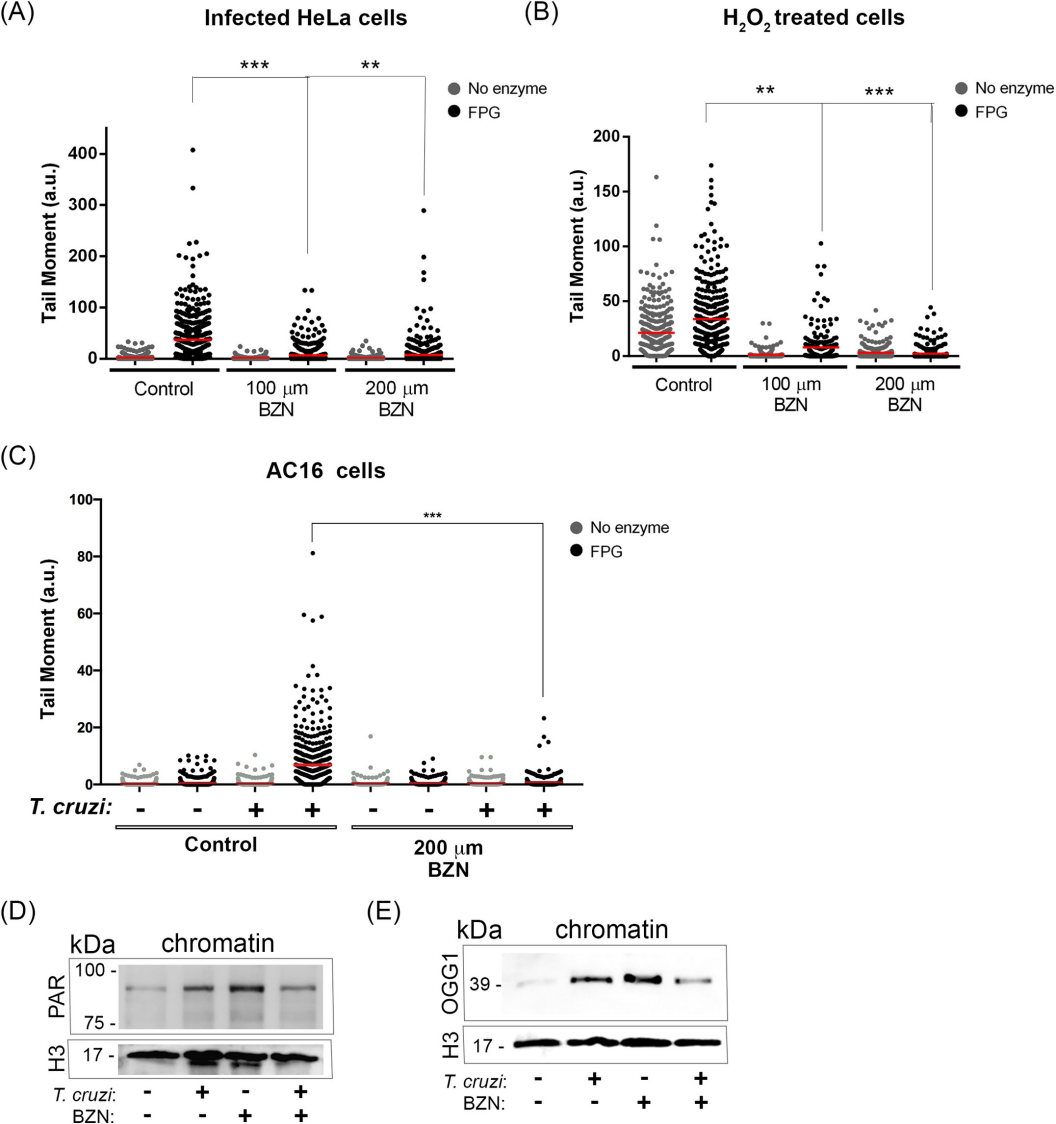
Benznidazole reduces DNA damage developing an adaptive response to HeLa cells. Comet assay was performed in (A) Infected HeLa cells, (B) cells treated with H_2_O_2_ (20 μm), or (C) Infected AC16 cells that were pretreated or not with 100 μm or 200 μm Benznidazole (BNZ). Scatter plots from tail moment quantification of 3 independent experiments (100 cells per experiment) were performed.**p<0.01; ***p<0.001. Isolated chromatin from infected cells or not that were pretreated or not with BNZ (200μm) were immunoblotted with (E) anti-PAR (F) anti-OGG1. Histone 3 was used as a loading control.

The effects of BNZ pretreatment were also investigated on DNA lesions in infected cells. Comet assays revealed that host cells pretreated with 100 or 200 μm of BNZ display less base oxidized DNA lesions when compared to the infected cells in the absence of BNZ after 1 h (Fig 5A). *T*. *cruzi* internalization was not affected by the presence of BNZ at the doses employed (100 and 200 μm) (S4 Fig). Thus, the reduction of DNA lesions is probably due to an effect of the drug in the host cell, not a secondary effect due to a trypanocide effect.

To test the BNZ effects on the host cells, control experiments were performed by treating (uninfected) cells with 20 μm H_2_O_2_ for 1 h (Fig 5B). Interestingly, pretreatment with BNZ (100 or 200 μm) reduces the induction of breaks and oxidized base DNA lesions in these cells. Also, AC16 infected cells pretreated with BNZ (200 μm) revealed a significant decrease in oxidized DNA lesions compared to infected cells without BNZ treatment (Fig 5C). Tail moment analyzed from cells only pretreated with BNZ (100 μm and 200 μm) not infected nor treated with H_2_O_2_ was insufficient to cause such an effect (S5 Fig).

Possible BNZ effects were also checked on DNA repair pathways. First, the effects of BNZ in the PARylation of chromatin from cells infected with *T*. *cruzi* were analyzed (Fig 5D). As expected, DNA from infected cells presented increased levels of PARylation, when compared to control cells. Interestingly, DNA PARylation levels were also high in uninfected host cells treated with BNZ. Infection with trypanosome in cells pretreated with BNZ reestablished PARylation levels nearly to the control not treated with the drug 1 h post-infection. The same profile was observed when OGG1 recruitment to the chromatin of cells was assessed (Fig 5E). Altogether, these data indicate that pretreatment of cells with BNZ may prepare host cells to receive the parasite and then solve DNA lesions faster than cells only infected.

## Discussion

Previous work has shown that ROS are generated by the loss of mitochondrial membrane potential during infection by *T*. *cruzi* [21]. As a direct result of this oxidative unbalance, DNA lesions (8-oxodG) are induced in the host cells, and there is an increase of total PARylation in infected cells [25]. Similar observations were made in myocardial tissue extracted from mice with chronic infection (150 days post-infection) [26]. The present study investigated the effects of *T*. *cruzi* on host cell DNA metabolism during different stages of infection. We went further and measured DNA lesions-derived oxidated bases at different time points. Therefore, we found that 1 h of infection is sufficient to induce a massive oxidated base lesions in HeLa cell and AC16 human cardiomyocyte cell line. It is well known that during the invasion, trypomastigotes entering the host cell require a cytosolic Ca^2+^ influx [27]. Although we did not measured directly this influx in the host cells, Ca^2+^ elevation in mammalian cells may affect several mitochondrial functions [28], including membrane potential loss, which leads to a peak in intracellular ROS that can be responsible for the early genotoxic effect observed in the present study. Interestingly, early infection of human primary cardiomyocytes results in increased respiratory capacity, as well as mitochondrial biogenesis [29]. Although this seems contradictory to the loss of mitochondrial potential, it may simply reflect that host cells are responding to the infection trying to recover from the initial damage on mitochondria. In fact, many of the deleterious effects (including DNA damage) and responses observed in the infected HeLa cells seem to recover at later times of infection.

Since we found an increase in oxidated DNA lesions 1 h post-infection, we also checked for other markers that could indicate that the host cell is dealing with an extreme genotoxic event. Indeed, we observed a correlation between oxidative DNA lesions and the levels of carbonylated proteins present in the chromatin. An increase in the amount of carbonylated proteins is considered a marker for oxidative stress during various pathological conditions such as atherosclerosis, aging, cancer, and neurodegenerative diseases [30]. However, this event has not yet been described in *T*. *cruzi* infection. The extensive oxidative DNA lesions that we found also correlated with the H2Ax phosphorylation. Though, we observed a discrete increment of phosphorylated H2Ax (γH2Ax) after 1 h of infection. H2Ax phosphorylation has been well demonstrated subsequently to double-strand break [31]. However, this event in response to oxidative burst may vary depending on the cell type and genotoxic agent [32]. Furthermore, a slight decrease in RNA synthesis was observed 1 h post-infected cells population. Thus, this decrease in RNA synthesis is not selective to infected cells, suggesting that *T*. *cruzi* could modulate not only cells that are being invaded, but also neibouring cells.

Although we have tested only one strain of *T*. *cruzi*, it is known that this parasite can sense and induce ROS in host cell differently according to Discrete Typing Unit (DTU). The present study used G strain which belongs to DTU I group. Remarkably, recent work has demonstrated that higher levels of oxidative species in cardiomyocytes occurred upon infection with a DTU I strain (JG strain) compared to DTU II strain [33]. Another important question for most of the effects observed at early times after infection is that only 20% are initially infected, so that the real effects could be underestimated. In fact, the results for DNA damage (FPG sensitive sites for example) by comet assay show very heterogeneous responses, with many cells (HeLa and AC16) not presenting lesions 1 h after infection. They could correspond to non-infected cells. However, evidences indicate that infected cells may secrete proteins, associated or not to vesicles, in the extracellular medium activating/modulating host cell responses [34–36]. Thus, we cannot exclude the possibility that even in the absence of productive infection, cells are under oxidative stress and suffering the effects from the 20% infected cells.

It is noteworthy that DNA lesions were still detected at 6 h after infection. Remarkably, other types of lesions were detected, such as single-strand breaks and alkaline sensitive sites. At this time point, we did not evidence γH2Ax compared to control cells. These data indicate that the host cell is beginning to solve DNA lesions at this time point. Indeed, our study searched for a specific PARP association with HeLa cell DNA lesioned by *T*. *cruzi*. PARP-associated chromatin from infected cells was increased compared to uninfected cells after 6 h of infection. However, we did not find significant differences in mRNA relative expression, indicating that PARP-1 mRNA transcription is a prior event. Another protein associated with the BER pathway is 8-oxoguanine glycosylase (OGG1). In our model, OGG1 appears to be increased only after 1 h of infection, and then it returns to basal levels 6 h later. Also, at 6 h infection, we found a decrease in OGG1 mRNA levels compared to the control in HeLa cells. This finding indicates that 8-oxoG accumulates in the DNA during 1 h of infection, which corroborates with the highest tail moments with the FPG enzyme found at this time point. Later on, the fact that OGG1 is not increased at 6 h post-infection and the reduction on the frequency of FPG sensitive sites suggests that the repair of these lesions (possibly by BER) is performed rapidly in infected cells. Higher levels of XRCC1 mRNA in HeLa cells and AC16 cells after 6 h of infection were observed. This protein is known to participate in the repair of single-strand breaks, and damage generated by alkylating agents, reactive oxygen species, or ionizing radiation [37]. XRCC1 also interacts with PARP-1 [38] and Polymerase β (POLβ) [39]. Interestingly, a previous study revealed that POLβ gene was upregulated at the early stages of infection [40]. Moreover, that study performed a transcriptome from infected HeLa cells at early stages and found upregulated different genes involved in mismatch DNA repair, direct repair, and BER pathways [40]. Altogether, data strongly suggest that different types of DNA damage are generated in the genome of the host cells, during parasite infection. Several DNA repair pathways are involved in process of protecting the host cell, with different kinetics of gene expression and function.

Considering the genotoxicity, mainly derived from oxidative stress during infection, we searched for proteins from the BER pathway. Previous work revealed that total cell PARylation was increased in infected cells 6 to 48 h post-infection. Recently, PARP-1 was shown to play an essential role in the chronic Chagas disease mouse model [26]. The authors evidenced that PARP1 KO mice display a reduction in parasite load and that PARP1/PAR affects mitochondrial integrity during infection. Our study revealed that PARP-1 accumulates in host chromatin 1 h, 6 h, and 72 h post-infection. Although the cell death mechanism is not well determined in *T*. *cruzi* infection, we found higher PARP levels at this final moment of infection could indicate that the host cell is dying by parthanatos. Accordingly, we found that DNA lesions from these time points (1 h, 6 h, and 72 h) were elevated compared to the other infection stages. Indeed, 24 h and 48 h post-infection, host cell DNA lesions are reestablished to control HeLa cells. This result indicates that the host cell is dealing with the genotoxic effect as infection progresses. However, DNA lesions return to increase 72 h post-infection. At this stage, parasites occupy the whole cytosol space from the host cell, and we observed an accumulation of higher tail moments that could represent genomic instability preceding cell death. When compared to infected cells after 48 h, this genotoxic event was accompanied by higher levels of γH2Ax and carbonylated protein. Interestingly, carbonylated proteins were concentrated in the 45–60 kDa range. Further study will be addressed to investigate why carbonylated proteins from host cell chromatin were found at this molecular weight range.

*T*. *cruzi*-induced cell death is still in debate. A previous study revealed that parasite infection could generate an anti-apoptotic effect in cardiac cells, with inhibition of caspases 3, 8, and 9 [41]. Further, another group showed the opposite effect of *T*. *cruzi* infection in the same cell line. They revealed that infected cells enter apoptosis with activation of caspases 3/7 and 8 [42]. It is well known that cell cycle arrest may precede apoptosis or other programmed cell death. In the present study, we investigated whether the infection could interfere with host cell replication at late stages (72 h). Therefore, cells containing parasites have an impairment in S-phase entry compared to cells that do not contain parasites in the infected population. Also, the impairment is higher, according to the parasite load. This data corroborates with the transcriptome from HeLa infected cells, which presented several proliferation inhibitor genes upregulated 72 h post-infection, and some proliferation inductors genes downregulated [43]. Previous work observed that cell cycle arrest occurs somewhere between 48 h and 72 h post-infection [44].

As mentioned above, oxidative stress plays an important role in *T*. *cruzi* infection. A previous study evidenced that parasites may benefit from the ROS elevation to multiplicate [22,45]. In HeLa cells, we observed that after 6 h of infection, NRF2 is more expressed and recruited to the host nucleus. This result suggests that NRF2 is signaling at this time point after a significant oxidative burst caused by *T*. *cruzi* during invasion. Later on, expression starts to decrease as well as the oxidative DNA lesions (24 h post-infection). Through parasite intracellular life cycle, NRF2 levels are reducing, and KEAP1 levels appear to be increasing. This result led us to assume that KEAP1 is probably leading NRF2 to degradation at the later stage of infection. NRF2 is being degraded, supporting the fact that parasites may benefit from the reduction in the antioxidant pathway to multiplicate and sustain infection.

Paiva et al. [22] observed that macrophage treatment with antioxidant NAC and NRF2 inductor (SFN) impairs *T*. *cruzi* intracellular life cycle in macrophages. In our study, parasite growth was also affected by the presence of NAC and SFN in a dose-dependent manner in HeLa cells. It is well characterized that *T*. *cruzi* presents several mechanisms to deal with oxidative stress and also specialized and efficient pathways to repair DNA damage [23,24]. Therefore, as already suggested before, oxidative stress may not only be beneficial for parasite growth. Yet, ROS exacerbation could be more deleterious to the host cell than to the parasite by leading to a genotoxic effect that can promote host cell cycle arrest and death.

Recent work demonstrated that BNZ stimulates NRF2 expression in non-infected hepatic cells [11]. NRF2 elevation was associated with an adaptative response to the increase in ROS induced by BNZ [10,11]. In our model, parasite growth was affected by BNZ treatment, as we observed with SFN, which induces NRF2 expression. Our study corroborated that BNZ acts in the host cell, and not only in the parasite, as we found that it protects DNA from oxidative stress in HeLa cells and AC16 cells. Remarkably, *T*. *cruzi* invasion was not affected by BNZ pretreatment. This finding was important to ensure that the effect observed was due to the host cell’s drug action and not due to an indirect effect of BNZ in parasite viability.

Furthermore, we checked whether BNZ could protect DNA lesions caused by H_2_O_2_ treatment. Notably, BNZ decreases mammalian cell DNA lesions derived from oxidative injury. This result led us to investigate whether BNZ could induce an adaptive response when infected with *T*. *cruzi*. The adaptive response is a mechanism in which a small conditioning dose causes a cell resistance when challenged with a higher dose [46]. Previous studies have reported that low doses of H_2_O_2_ induce a protective response when the cells are submitted to higher doses of H_2_O_2_ [47–49]. Moreover, it was shown that this preliminary challenge with lower doses of H_2_O_2_ upregulated DNA repair genes [49]. The adaptive response could explain BNZ treatment’s effect in DNA PARylation, and the OGG1 association in control cells was much higher than the infected cells. In other terms, BNZ generated previous oxidative stress that may have led to an earlier PARylation, recruitment of OGG1, and activation of NRF2 to solve DNA lesions. Therefore, when cells that were previously treated with BNZ are infected, they are already prepared, and the answer to repair DNA lesions is much faster compared to cells that were not treated with BNZ. BNZ could probably trigger an adaptive response that protects mammalian cells from oxidative damage caused by *T*. *cruzi*.

In this study, we have characterized the development of genotoxicity induced by *T*. *cruzi* in HeLa cells and cardiomyocytes. Altogether, the parasite alters DNA metabolism from the host cell and may benefit from this fact to multiplicate and disseminate infection. Also, BNZ pretreatment appears to have a preventive effect on the host cell, which could be important for further studies. In our perspective, the parasite benefits from oxidative stress, yet the host cell has more to lose than the parasite.

## Material and methods

### Cell cultures and parasites

HeLa cell line (Instituto Adolpho Lutz) and AC16 human cardiomyocyte cell line (Millipore) were used to investigate host cell DNA metabolism in the *Trypanosoma cruzi* infection. Vero cells (Instituto Adolpho Lutz) were used to maintain *T*. *cruzi* intracellular life cycle *in vitro*. These cells were grown on RPMI-1640 (LGC Biotechnology) supplemented with 10% fetal bovine serum (FBS; Gibco) and kept in at 37°C and 5% CO_2_. We used *T*. *cruzi* G strain (DTU Tc I) (Yoshida, 1983). Cell culture-derived trypomastigotes (TCTs) were obtained from the supernatant of infected Vero cells and used in all the experiments described below.

### *T*. *cruzi* infection assay

HeLa cells or AC16 cells were plated onto different types of plates according to the experiment and kept overnight at 37 ^o^C and 5% CO_2_ environment. Then, TCT’s were incubated with HeLa cells in RPMI 10% FBS medium in the proportion of 20:1 (parasite:cell). Cells were incubated at 37 ^o^C with 5% CO_2_ for at least 1 h. After that, cells were washed with PBS to remove parasites that have not been internalized. For early time points (1 h and 6 h), infected cells were maintained in RPMI 10% FBS medium. For later time points (24 h, 48 h, and 72 h), cells were incubated with RPMI 2.5% FBS for better parasite growth.

Additionally, cells were pretreated with 100 μm or 200 μm Benznidazole (BNZ; Sigma-Aldrich) and incubated for 16 h at 37 ^o^C and 5% CO_2_, and then cells were infected with *T*. *cruzi* for 1 h. Next, comet assay and western blotting from isolated chromatin were performed. For comet assay, instead of infecting HeLa cells with *T*. *cruzi*, cells were treated with H_2_O_2_ (20 μm) as a positive control of oxidative stress.

### Quantification of intracellular *T*. *cruzi* amastigotes

For quantifying intracellular parasite growth, HeLa cells adhered to glass coverslips were placed in a 12-well plate and then infected as described above. After the infection period, cells were washed with PBS and incubated at 37 ^o^C with RPMI 2.5% SBF in the presence or absence of different concentrations of N-acetyl cysteine (NAC; Sigma-Aldrich), Sulforaphane (SFN; SigmaAldrich), or Benznidazole (BNZ; Sigma-Aldrich). Parasite growth was evaluated 48 h later, and cells were fixed with Bouin (Sigma-Aldrich) for 5 min and stained with Giemsa (Sigma-Aldrich) for 1 h. Counting was performed in an optical microscope of parasites internalized in 100 infected cells per coverslip.

### Immunofluorescence

Infected cells attached to 13 mm coverslips were with PBS and fixed with paraformaldehyde (PFA) 4% for 15 min at room temperature. Then, fixed cells were washed with PBS and incubated with a PGN blocking solution (0.2% gelatin and 0.1% sodium azide) for 1 h. Subsequently, cells were incubated for 16h with primary anti-γH2AX antibody (1:500, Millipore) or anti-PARP1 (1:100; Abcam; ab6079) diluted in PGN solution with 0.1% Saponin. Next, cells were incubated with Alexa Fluor 594 or Alexa Fluor 488 (Molecular Probes;1:100) for 1 h in PGN-saponin solution in the presence of 10 μM 4’,6-diamino-2-phenylindole hydrochloride (DAPI; Molecular Probes). Finally, coverslips were mounted in glycerol buffered with 0.1 M Tris pH 9.0 and p-phenylenediamine 0.1%. Slides were examined and analyzed in Axiovert 200 (Carl Zeiss) fluorescence microscopy. γH2Ax fluorescence quantification was performed using ImageJ (v. 2.1.0). Foci from at least 100 cells in triplicate were analyzed using Integrated density (IntDen) values.

### Incorporation of bromodeoxyuridine (BrdU)

Cells were plated on glass coverslips, and infection was followed as previously described. After 72 h of infection, cells were treated with 10 μM Bromodeoxyuridine (BrdU) for 30 min at 37 ^o^C. The cells were then fixed with 4% paraformaldehyde (PFA) for 15 min at room temperature. After fixation, cells were treated with 1 M HCl for 30 min, and immunofluorescence was performed as described above. In these experiments, the primary anti-BrdU antibody (Thermofisher) was used.

### Gel electrophoresis and western blotting

Protein extract (20 μg) was applied on 10% polyacrylamide gel. Later, proteins in the gel were transferred to a nitrocellulose membrane for Western blotting (Towbin et al., 1979). The membrane was blocked for 1 h with 5% bovine serum albumin (BSA; Sigma-Aldrich) at room temperature and subsequently incubated with the primary antibody PBS-T (PBS, 0.1% tween) with 5% BSA (Sigma-Aldrich), for 16 h at 4 ^o^C. The primaries antibodies used were anti-PARP1 (1:1000; Abcam; ab6079), anti-γH2Ax (1:1000; Cell Signaling), anti-NRF2 (1:500; Abcam), anti-p-NRF2 (1:1000; Abcam), anti-OGG1 (1:1000; Abcam), Anti-pan-ADP-ribose binding reagent (1:1000; Merck) and anti-histone 3 (Abcam; 1:5000). Then, membranes were placed in PBS-T solution with 5% milk, and secondary anti-mouse or rabbit IgG peroxidase antibody (Sigma-Aldrich) diluted 1:5000. The membrane was developed with chemidoc (UVITEC) in the presence of Amersham ECL Prime Western Blotting Detection Reagent (GE Healthcare). Alternatively, membranes were stripped with Restore Western Blot Stripping Buffer (ThermoFisher) and reincubated with another primary and secondary antibody. Band quantification was performed in ImageJ (v 2.1.0). Briefly, the area from relative density of each band from target protein was measured and normalized with endogenous control.

### Carbonylated proteins

Carbonylated proteins were investigated in chromatin from infected cells and not infected. First, the cells were denatured, as described previously [50]. Next, proteins were submitted to gel electrophoresis and Western blotting. The primary anti-DNPH antibody (1:1000, Sigma-Aldrich) was used to reveal carbonylated proteins, followed by incubation with secondary anti-rabbit peroxidase antibody (1:5000, Sigma-Aldrich).

### Purification of chromatin-associated proteins

Cells (3x10^6^) grown in 150 cm^2^ plates were infected, as described above. After infection, cells were trypsinized and centrifuged at 1,000xg for 5 min. Then, we proceeded to chromatin-associated protein purification [51]. Briefly, pelleted cells were lysed by adding solution A (250 mM sucrose, 1 mM EDTA, 3 mM CaCl_2_, 10 mM Tris-HCL pH 7.4 and 0.5% saponin), and centrifuged at 3,000xg for 10 min at 4°C. Then, pellets were resuspended following the same procedure and centrifugation with solution B (250 mM sucrose, 1 mM EDTA, 3 mM CaCl_2_, 10 mM Tris-HCL pH 7.4). Next, pellets obtained in this step (corresponding to the nuclear fraction) were resuspended with solution C (1% Triton X100, 15 mM NaCl and 25 mM EDTA and 10 mM Tris-HCL pH 8) was added and homogenized centrifuged at 12,000xg for 20 min at 4°C (step corresponding to obtaining the chromatin). Pellets were then washed 3 times with 10 mM Tris-HCl pH 8.0, centrifugation at 12,000xg for 20 min at 4°C. Chromatin extracts were sonicated 3 x 30 s output 7, Duty 70 in the Tomy ultrasonic disruptor sonicator UD-201, with a 1 min interval between sonications. Samples were quantified with the BCA protein quantification kit (TheroFisher) and analyzed by Western blotting.

### Comet assay in cells infected with *T*. *cruzi*

The comet assay’s detection of breaks in genomic DNA in infected cells was carried out as previously described [52]. Infected cells at different timepoints were trypsinized and resuspended in 0.5% low melting agarose at 37 ^o^C and spread evenly across microscopy slides covered with 1.5% agarose. Slides were incubated for 10min at 4°C to solidify low agarose melting. The samples were then incubated in a lysis solution (2.5 M NaCl, 100 mM EDTA, 10 mM Tris, 1% Triton X-100, and 10% DMSO at pH 10.0) for 16 h at 4 ^o^C. After lysis the cells were treated with the enzyme formamidopyrimidine-DNA E. coli glycosylase (FPG; BioLabs) diluted in enzyme buffer (0.1 M KCl, 0.5 mM Na_2_EDTA, 40 mM HEPES, 0.2 mg/ml BSA, pH 8.0). Then, the blades were incubated in electrophoresis well with an alkaline solution (300 mM NaOH and 1 mM EDTA, pH 13.0), and they were subjected to electrophoresis (300 mA and 25 V) by 20 min. The slides were neutralized with 0.4 M Tris-HCl buffer (pH 7.4) and fixed with 100% ethanol. The slides were stained with 20 μg/mL bromide of ethidium solution (Sigma-Aldrich). The head/tail ratio was quantified to 100 cells per slide using the LUCIA Comet Assay program (Laboratory Image).

### Flow cytometry for γH2Ax quantification

Cells were trypsinized and fixed for 15 min on ice in a 1% formaldehyde solution (diluted in PBS). The cells were then fixed with ethanol 70% and incubated at -20°C for at least 24 h. Subsequently, samples were resuspended in PBS-T-BSA solution (PBS; 0.2% Triton-X-100 and 1% BSA) and blocked for 5 min at room temperature. The samples were then incubated with primary anti-γH2AX antibody (1:500; Merck-Millipore) in PBS-T-BSA and maintained for 16 h at 4 ^o^C. Next, cells were incubated for 1 h at room temperature with anti-mouse FITC antibody (1:200; Sigma-Aldrich) in PBS-T-BSA solution. Finally, cells were washed with PBS-T-BSA and incubated for 1 h at room temperature in sodium propidium iodide solution (PI; 200 mg/ml RNase; 20 mg/ml PI; 0.1% Triton X-100 in PBS). For analysis on the FACS BD Accuri C6 cytometer, cells were washed to remove PI and resuspended in PBS.

### EU incorporation

Infected cells or uninfected cells were incubated with 1 mM of 5-Ethynyl Uridine (EU; Invitrogen), uridine analog, and incubated for 1 h with RPMI 10% medium at 37 ^o^C and 5% CO_2_. Next, cells were fixed with 1% formaldehyde for 15 min at 4 ^o^C, and then with 70% ethanol at 20 ^o^C overnight. Subsequently, cells were precipitated and washed in a solution of PBS-T-BSA. After centrifugation, cells were permeabilized and blocked with PBS-T-BSA for 5 min at room temperature. Next, samples were resuspended and incubated for 30 min at room temperature, in the dark, in a solution with Click-iT RNA Alexa Fluor 488 HCS Assay kit (Invitrogen) according to the manufacturer’s recommendations. After that, the samples were washed with PBS, centrifuged, and resuspended in PBS in the desired volume. For the analysis, the FACS BD Accuri C6 used the counting of 20 thousand events and the evaluation of the average fluorescence. Alternatively, cells incubated with the EU were fixed onto coverslips, fixed with 4% PFA. Next, coverslips were incubated with the same Click-IT solution and visualized by a fluorescence microscope.

### Real-time PCR

Initially, RNA was extracted from infected or uninfected HeLa cells with the RNeasy Protect Mini kit (Qiagen). Then, the cDNA was synthesized from these RNAs using the High Capacity DNA Reverse Transcription kit (Applied Biosystems). For each reaction, 25 ng cDNA, 300 nM primer forward, and 300 nM of reverse primer diluted in SYBR Select MasterMix (Applied Biosystems). The primers used amplified a specific region (approximately 100 bp) of the PARP1, OGG1, XRCC1, NRF2 and KEAP1 genes. The qPCR was performed in StepOne Plus equipment (Applied Biosystems). Thus, in the qPCR reaction, constant monitoring of the fluorescence emitted in each well by the intercalation of the SYBR @ Select Green MasterMix compound (Applied Biosystems) in double-stranded DNA. The normalized values were calculated using the formula ΔΔCycle Threshold (Ct) and entered in a data sheet for representation print shop. As a normalizer of the experiment, primers were used that amplify a 100 bp region of ACTB, a gene constitutively expressed in HeLa.

### Statistic analysis

All results are representative of three independent experiments with at least two biological replicates. GraphPad Prism 4.0 was employed for data plotting and statistical analysis. Statistical tests included Student’s t-test, ANOVA or non-parametric statisc tests. Statistical p < 0.05 was used as the threshold.

## Supporting information

S1 Fig *P*hosphorylation of H2Ax 6 h post-infection in cardiomyocytes is similar to control cells. (A) Human cardiomyocytes AC16 cell line infected with *Trypanosoma cruzi* (+; MOI:20:1) or not (-) were fixed with 4% Paraformaldehyde (PFA). Next, cells were incubated with anti-γH2Ax and stained with secondary antibody Alexa Fluor 488 (green). Nuclei were stained with DAPI (blue). Scale bar: 10 μm. (B) Quantification of Integrated Density (fluorescence) was performed with ImageJ. The bar graph represents the mean and standard error of the mean (SEM) of Integrated Density (IntDen) from at least 100 cells per sample from 3 independent experiments.
(TIF)

S2 Fig Synthesis of newly produced RNA is reduced by infection with *Trypanosoma cruzi*. HeLa cells (A) not infected and (B) infected were incubated with 5-ethynyl uridine (EU) for 1 h. Next, cells were fixed and stained with an EU-binding probe (green). Nuclei were stained with DAPI (blue). Scale bar: 10 μm.
(TIF)

S3 Fig DNA repair mRNA relative expression from infected cardiomyocyte cells. AC16 cells were infected or not with *Trypanosoma cruzi* (MOI 20:1). mRNA from Infected cells and control was extracted 6 h post-infection. Next, from RNA samples, cDNA was synthesized for Real-Time PCR reaction. Graphs show the mean and standard deviation from 3 independent experiments of relative expression (ddCT) from gene*s PARP-1*, *OGG1* and *XRRC1* from infected cells compared to control 6 h post-infection. *ACTB* gene was used as the endogenous control. ***p<0.001.
(TIF)

S4 Fig *Trypanosoma cruzi* invasion is not altered in HeLa cells treated with Benznidazole at lower doses. HeLa cells pretreated with Benznidazole (100 μm, 200 μm, and 400 μm) for 16 h, control (-) were not treated. Later, cells were infected with trypomastigotes (MOI: 20:1) for 1 h. Next, cells were fixed and stained with Giemsa. Internalized parasites from 100 cells were counted. Graphs shows mean and standard deviation from 3 independent experiments performed in duplicate. **p<0.01; ns: not significant.
(TIF)

S5 Fig Benznidazole does not induce DNA damage to control HeLa cells. Comet assay was performed in HeLa cells only treated with Benznidazole (BNZ) for 16 h. After treatment with BNZ (100 μm and 200 μm). Next, cells nuclei were treated with Formamidopyrimidine DNA glycosylase enzyme (FPG; black dots) or not (No enzyme; grey dots). To analyze the DNA strand breaks, cells nuclei were stained with ethidium bromide and visualized with a fluorescence microscope. (A) Scatter plots from tail moment quantification of 3 independent experiments (100 cells per experiment) were performed. ns: not significant.
(TIF)
