# Supplementary figures and images for: DNA damage and oxidative stress in human cells infected by Trypanosoma cruzi
Source: PLoS Pathog. 2021 Apr 7;17(4):e1009502. doi: 10.1371/journal.ppat.1009502 (PMC8087042; doi:10.1371/journal.ppat.1009502)

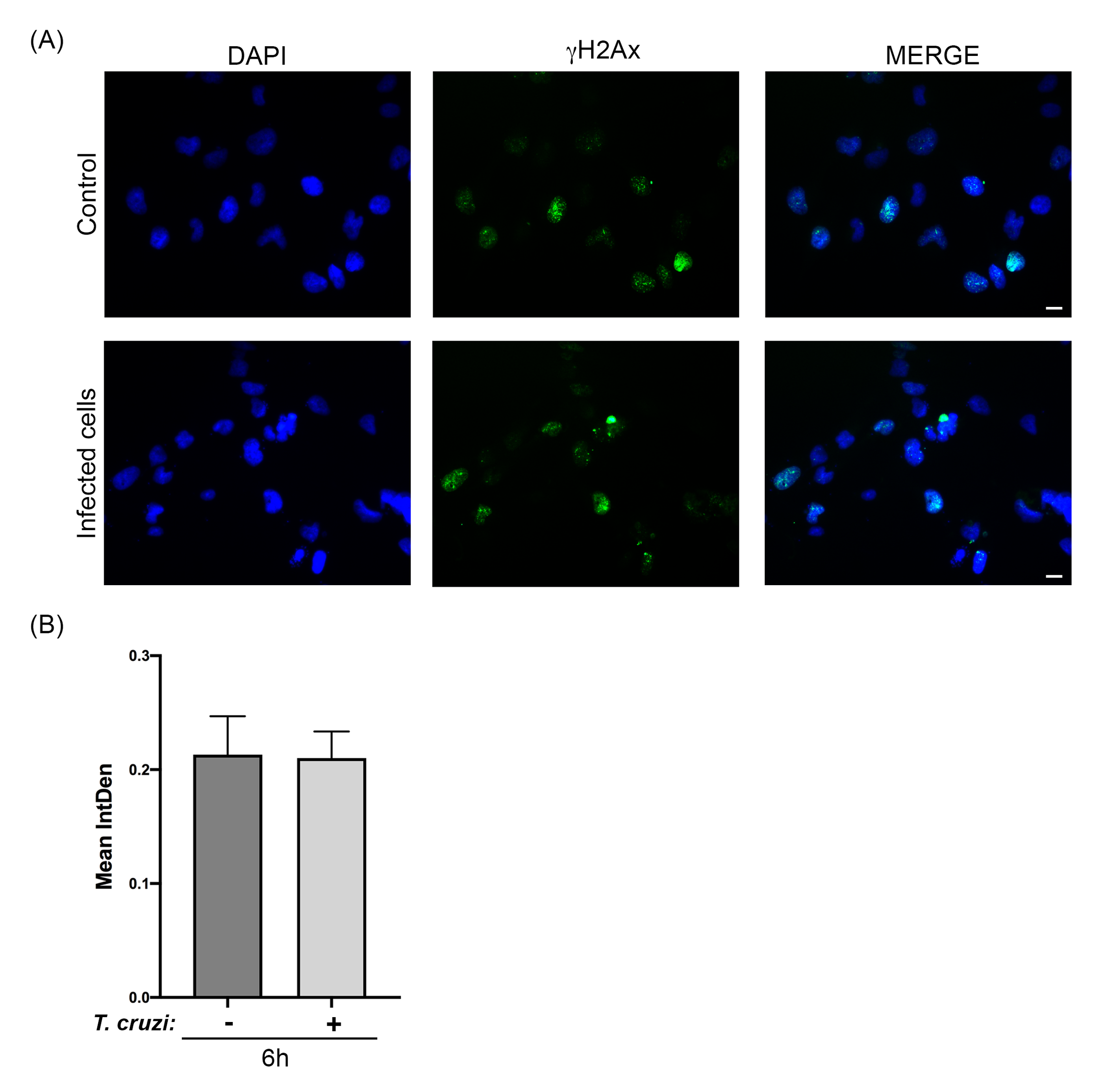

Supplement: S1 Fig — (A) Human cardiomyocytes AC16 cell line infected with Trypanosoma cruzi (+; MOI:20:1) or not (-) were fixed with 4% Paraformaldehyde (PFA). Next, cells were incubated with anti-γH2Ax and stained with secondary antibody Alexa Fluor 488 (green). Nuclei were stained with DAPI (blue). Scale bar: 10 μm. (B) Quantification of Integrated Density (fluorescence) was performed with ImageJ. The bar graph represents the mean and standard error of the mean (SEM) of Integrated Density (IntDen) from at least 100 cells per sample from 3 independent experiments. (TIF) [file ppat.1009502.s001.tif]

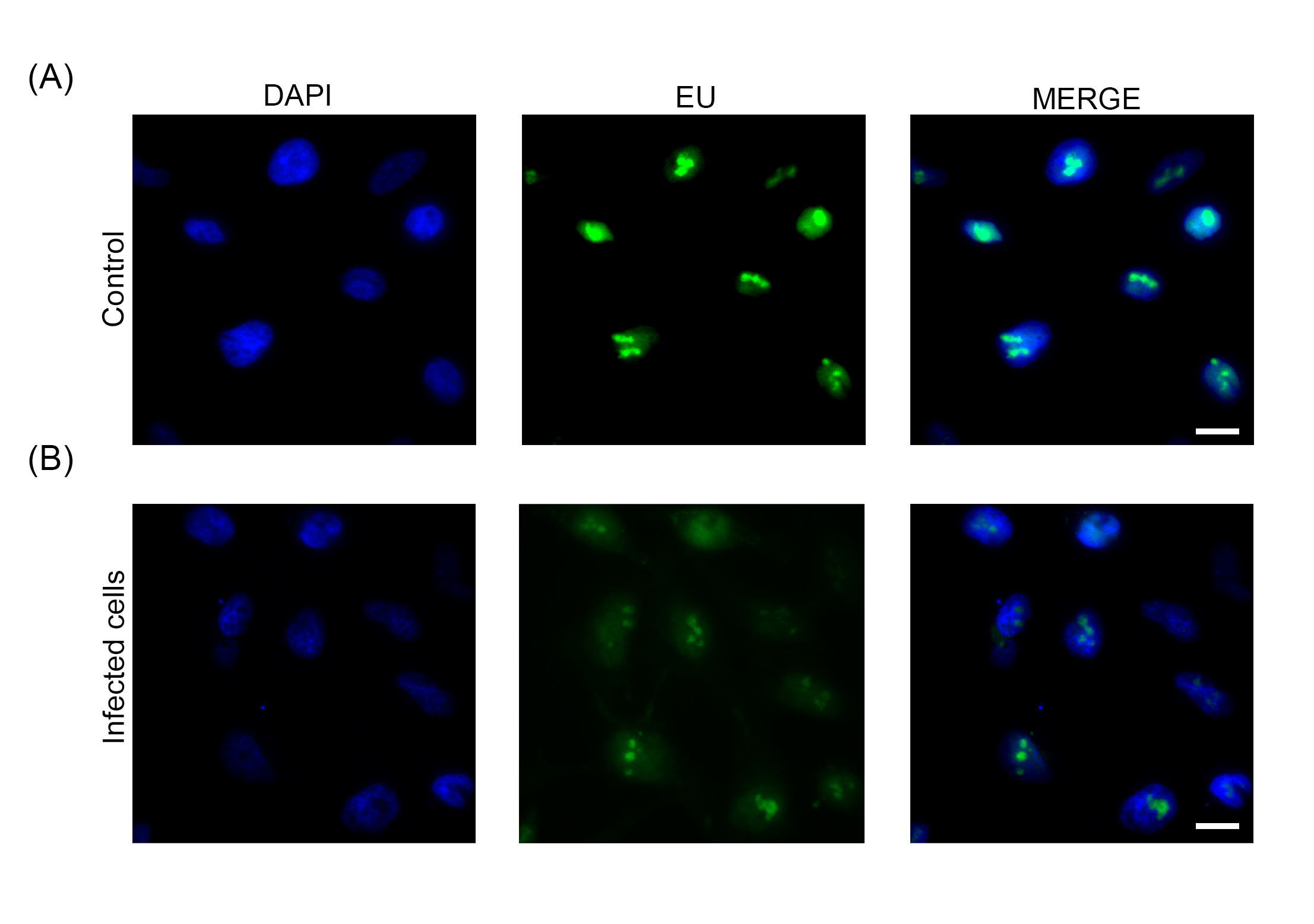

Supplement: S2 Fig — HeLa cells (A) not infected and (B) infected were incubated with 5-ethynyl uridine (EU) for 1 h. Next, cells were fixed and stained with an EU-binding probe (green). Nuclei were stained with DAPI (blue). Scale bar: 10 μm. (TIF) [file ppat.1009502.s002.tif]

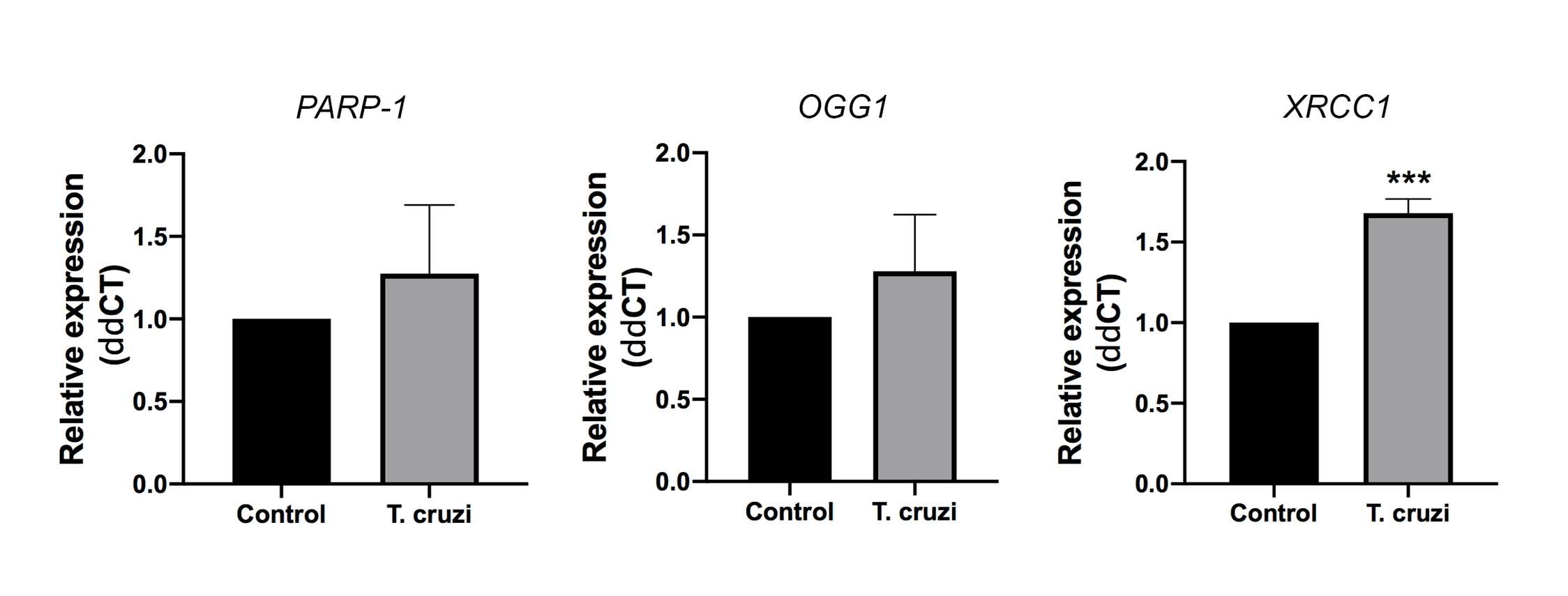

Supplement: S3 Fig — AC16 cells were infected or not with Trypanosoma cruzi (MOI 20:1). mRNA from Infected cells and control was extracted 6 h post-infection. Next, from RNA samples, cDNA was synthesized for Real-Time PCR reaction. Graphs show the mean and standard deviation from 3 independent experiments of relative expression (ddCT) from genes PARP-1, OGG1 and XRRC1 from infected cells compared to control 6 h post-infection. ACTB gene was used as the endogenous control. ***p<0.001. (TIF) [file ppat.1009502.s003.tif]

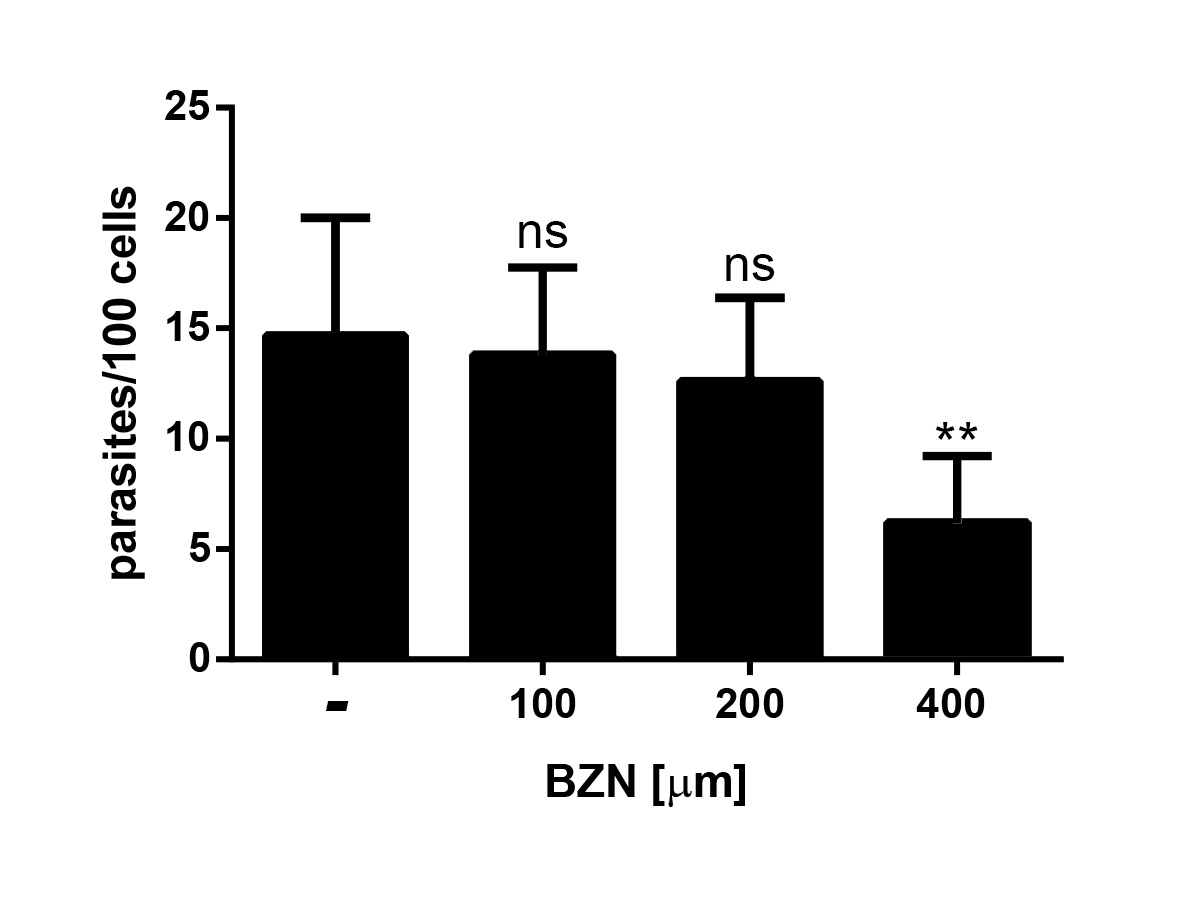

Supplement: S4 Fig — HeLa cells pretreated with Benznidazole (100 μm, 200 μm, and 400 μm) for 16 h, control (-) were not treated. Later, cells were infected with trypomastigotes (MOI: 20:1) for 1 h. Next, cells were fixed and stained with Giemsa. Internalized parasites from 100 cells were counted. Graphs shows mean and standard deviation from 3 independent experiments performed in duplicate. **p<0.01; ns: not significant. (TIF) [file ppat.1009502.s004.tif]

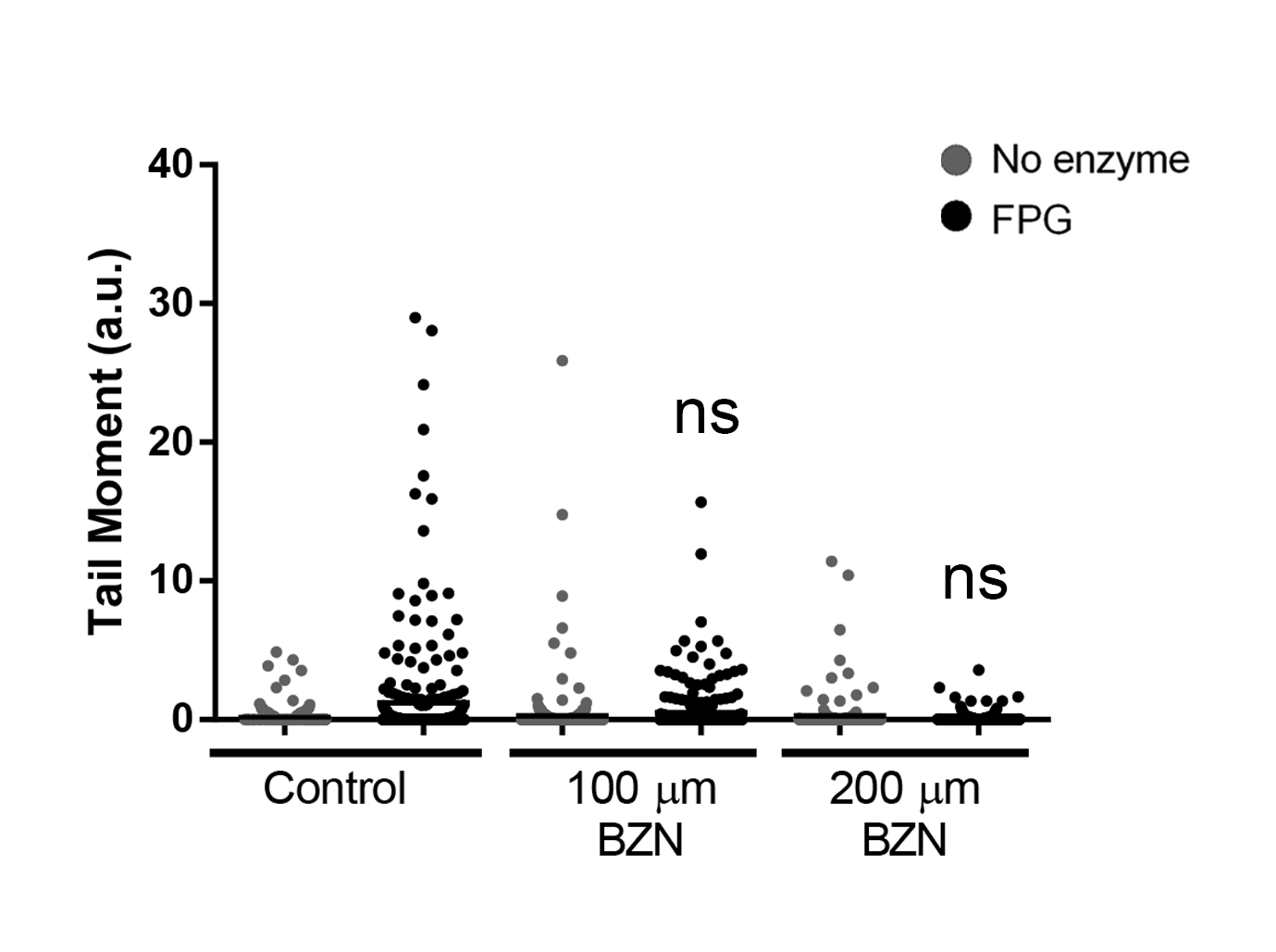

Supplement: S5 Fig — Comet assay was performed in HeLa cells only treated with Benznidazole (BNZ) for 16 h. After treatment with BNZ (100 μm and 200 μm). Next, cells nuclei were treated with Formamidopyrimidine DNA glycosylase enzyme (FPG; black dots) or not (No enzyme; grey dots). To analyze the DNA strand breaks, cells nuclei were stained with ethidium bromide and visualized with a fluorescence microscope. (A) Scatter plots from tail moment quantification of 3 independent experiments (100 cells per experiment) were performed. ns: not significant. (TIF) [file ppat.1009502.s005.tif]
